# Transfusion related acute lung injury with massive pulmonary secretion during cardiac surgery. A case report

**DOI:** 10.1186/1749-8090-9-64

**Published:** 2014-04-02

**Authors:** Julien Teodori, Kamal Rampersad, Giovanni Teodori, Roland Roopchand, Gianni Davide Angelini

**Affiliations:** 1 Caribbean Heart Care Medcorp, Port Of Spain, Trinidad and Tobago; 2 NILH Imperial College London, London, UK

**Keywords:** TRALI, Cardiac surgery, Acute lung injury

## Abstract

A Indo-Caribbean patient undergoing cardiac surgery developed Transfusion Related Acute Lung Injury (TRALI) with massive endobronchial secretion of clear fluid mimicking severe pulmonary edema. Hypoxemia and lung stiffness were so severe that didn’t allow closure of the sternum on completion of surgery. The patient was treated with invasive ventilation, high positive pressure and % FiO2 and aggressive endotracheal suction. After several hours, secretions reduced spontaneously and the patient made an uneventful recovery.

## Background

Transfusion Related Acute Lung Injury is a rare complication. The clinical pattern shows hypoxemia with bilateral pulmonary infiltrations, producing large amount of secretion mimicking cardiogenic pulmonary edema. Treatment is usually supportive with invasive ventilation. Herein we describe a patient with TRALI syndrome who was undergoing elective coronary artery bypass Surgery (CABG) and the strategy adopted for its management.

## Case presentation

A 72 year-old Indo-Caribbean man with three-vessel coronary artery disease underwent elective coronary artery bypass surgery on the beating heart with left internal mammary to the left anterior descending coronary artery and a saphenous vein graft to the posterior descending coronary artery.

Surgery was uneventful and the patient returned to the intensive care unit in stable condition. Two hours later, however, due to sudden bleeding and cardiac arrest, he required resuscitation and re-exploration. In order to control massive bleeding from a tear of the aorta at the proximal venous anastomosis and the persistent depressed cardiac function, it became necessary to establish cardiopulmonary bypass. The hemoglobin (Hb) before re-exploration was 4.5 mg/dl. The tear in the aorta was repaired, and the venous graft re-implanted higher in the Ascending Aorta. Transfusion of 6 Units of packed red cells (PRC) and 3 Units of fresh frozen plasma (FFP) was required to achieve Hb of 8.8 mg/dl at the end of surgery. Weaning from cardiopulmonary bypass was achieved with no inotropic support; however, it was difficult to ventilate the patient. The lungs were very stiff and distended. Despite of high positive pressure and % FiO2 the blood saturation remained very low. This was soon followed by copious pulmonary secretion requiring bronchial suction (around 100 ml of fluid collected each time), every 5 to 10 minutes. The fluid was clear, yellowish, and not frothy. The total amount of collected bronchial fluid before transfer to ICU was over 1700 ml. An IABP was inserted, but any attempt to close the chest resulted in significant decrease in the systemic arterial pressure. The patient was therefore return to the intensive care unit with the chest open.

Repeated bronchial suctions were required for about five hours before improvement in oxygen saturation. Bronchial secretion reduced and then suddenly stopped two hours after transfer to the ICU. Oxygenation improved and it was possible to close the chest the following day. Respiratory distress remained and the patient was extubated on day 5 postoperatively. He made a slow but uneventful recovery and was discharge home on day 20.

## Discussion

Transfusion related acute lung injury (TRALI) is a syndrome defined by acute pulmonary edema after transfusion of any blood component [1] Suspect TRALI is defined as fulfilmentof the definition of acute lung injury occuring within 6 hours of transfusion, in the absence of the other risk factors [2].

TRALI is considered one of the major causes of transfusion-related morbidity with ABO incompatibility and bacterial contamination, and the first cause of death in U.S. [3]. Its incidence is similar in both sex and at any age and is about 1 in 5000 transfusions [4]. The incidence is probably underestimated due to lack of application of diagnostic criteria.

The clinical pattern is dominated by acute respiratory distress syndrome (ARDS). TRALI can be differentiated from other forms of ARDS as invasive monitoring shows normal intracardiac pressures. Lung damage is generally transient and PO2 levels return to pre-transfusion levels within 48–96 hours. The symptoms may occur during 6 hours after transfusion, showing dyspnoea, tachypnea, hypotension and cyanosis [5]. Clinical exam shows severe pulmonary distress but no signs of heart failure or volume overload, confirmed by CXR analysis.

Treatment of TRALI is supportive with oxygen therapy in the mild forms, and with mechanical ventilation in the severe forms. Further blood product transfusion is strongly discouraged. There is no evidence of benefit with diuretics of corticosteroids [6]. Recovery is complete within 96 hours with no sequelae in the majority of the patients. A small percentage may present persistent pulmonary infiltrates up to seven days, and about 10% of cases have fatal outcome despite aggressive support [7].

The pathophysiology of TRALI is based on activation of neutrophil granulocytes at the onset of lung injury. These are trapped in the pulmonary microcirculation and oxygen free radicals and protheolitic enzymes are released. Pulmonary capillary leak syndrome develops with exudation of fluids and proteins. There are three main theories supported in literature: the first theory suggests the role of donor antibodies against recipient leukocytes causing complement and neutrophil activation with subsequent endothelial damage. This theory is supported by the presence of donorderived antibodies to HLA class I antigens and neutrophils in up to 89% of TRALI cases. The second theory is a “two hit hypothesis” where the first hit is an underlying condition of the patient with stimulation of adherence of neutrophils to the pulmonary epithelium. The second hit is associated with transfusion with reaction between antibody of the recipient and surface antigens on RBCs donor causing immediate degranulation of neutrophils [8]. Cardiopulmonary Bypass used in cardiac surgery with its well-known systemic inflammatory response can be considered as part of the “first hit”. The third hypothesis justifies the presence of this syndrome also in neutropenic patients by suggesting direct lung damage with endothelial fenestration, caused by the presence of high levels of vascular endothelial growth factor (VEGF) or antibodies to class II HLA antigens in donor’s blood [9].

Prevention for this syndrome is difficult. The main procedures able to reduce the incidence of TRALI are the use of predominantly male plasma for preparation of high-volume plasma components and pooling of plasma products [10]. Other strategies planned are to reduce the use of plasma from donors at high risk for HLA immunization (i.e. previously pregnant females) and not to transfuse patients at risk (i.e. those in severe clinical condition) with RBCs stored longer than 14 days or platelets stored longer than 2 days.

## Conclusions

Transfusion Related Acute Lung Injury (TRALI) is a rare but potentially lethal complication after heart surgery. This case report highlights the need for conservative management with mechanical ventilation of the lungs, frequently repeated suction of secretion from the endotracheal tube and avoidance of further transfusions. Leaving the chest open facilitates lung ventilation and minimises cardiovascular impairment.

## Consent

Written informed consent was obtained from the patient for publication of this case report. A copy of the written consent is available for review by the Editor-in-Chief of this journal.

The case description was performed in accordance to the latest Declaration of Helsinki.

## Abbreviations

TRALI: Transfusion related acute lung injury; CABG: Coronary artery bypass graft; Hb: Haemoglobin; PRC: Packed red cells; IABP: Intra aortic balloon pump; ICU: Intensive care unit; ARDS: Acute respiratory distress syndrome; CXR: Chest X Ray; RBC: Red blood cells; VEGF: Vascular endothelial growth factor.

## Competing interests

The authors declare that they have no competing interests.

## Authors’ contributions

JT collected data and drafted the manuscript. KR and RR were the anestethists of the case and contributed in the draft. GT was the surgeon of the case and conceived the the case report. G.D.A. participated in the draft of the manuscript and editing. All authors read and approved the final manuscript.
